# Investigating the differences in basic cardiopulmonary resuscitation between drowned people at sea and other people who need resuscitation

**Published:** 2023-12

**Authors:** Arman Zarbizadeh, Nooshin Babaei, Sima Eskandari

**Affiliations:** ^*a* Department of Operating Room, Faculty of Nursing, Baqiyatullah University of Medical Sciences, Tehran, Iran. ^; ^*b* Shahid Beheshti University of Medical Sciences, Tehran, Iran. ^; ^*c* PhD Student in Educational Psychology, North Tehran Branch, Islamic Azad University, Tehran, Iran. ^

## Abstract

**Background:**

Performing prompt basic life support is one of the essentials of helping drowning victims. This study aimed to investigate the difference between basic cardiopulmonary resuscitation in seawater-drowned victims and other victims needing resuscitation.

**Methods:**

The databases available in the National Digital Medical Library of Iran (Elsevier, and Nursing index), and Persian databases (Magiran, and SID) were searched for the keywords “drowning”, “cardiopulmonary” “resuscitation” and ”sea”, separately, and combined.

**Results:**

In-water resuscitation is only possible if it is performed by a highly skilled rescuer with only ventilation (no massage). Drowning people with only stopped breathing usually respond by giving a few rescue breaths. If the person does not respond to ventilation, the victim should be considered as having a cardiac arrest and quickly transferred to land for CPR and placed in a supine position with the head and trunk on the same level (usually parallel to the shoreline). If the person is breathing but not conscious, he should be placed in the recovery position (lateral decubitus). If the drowned cannot breathe, rescue ventilation is necessary. Unlike primary cardiac arrest, drowning can cause a ragged breathing pattern despite having a heartbeat, so only ventilation is needed. Cardiac arrest due to drowning occurs mainly due to lack of oxygen and CPR must follow the traditional ABC sequence (Airway-Breathing-Circulation), not CAB. The rescuer performs 5 initial rescue breaths, then 30 chest compressions, and continues with 2 rescue breaths and 30 chest compressions until vital signs reappear, the rescuer is exhausted, or advanced life support is available.

**Conclusions:**

Instead of 2 initial rescue breaths, 5 breaths should be given because it is more difficult to perform ventilation at the beginning due to the entrance of water into the airways with the effective opening of the alveoli. CPR with only chest massage is not recommended under one condition i.e. drowning.

**Keywords:**

Drowning, Cardiopulmonary resuscitation at sea, Cardiopulmonary resuscitation

